# Supplementary material for: Ethylene industrial emitters seen from space
Source: Nat Commun. 2022 Oct 28;13:6452. doi: 10.1038/s41467-022-34098-8 (PMC9616823; doi:10.1038/s41467-022-34098-8)
Supplement: Supplementary file 1 — Supplementary Information [file 41467_2022_34098_MOESM1_ESM.pdf]

Ethylene industrial emitters seen from space

By Franco, B. *et al.*

Supplementary Information

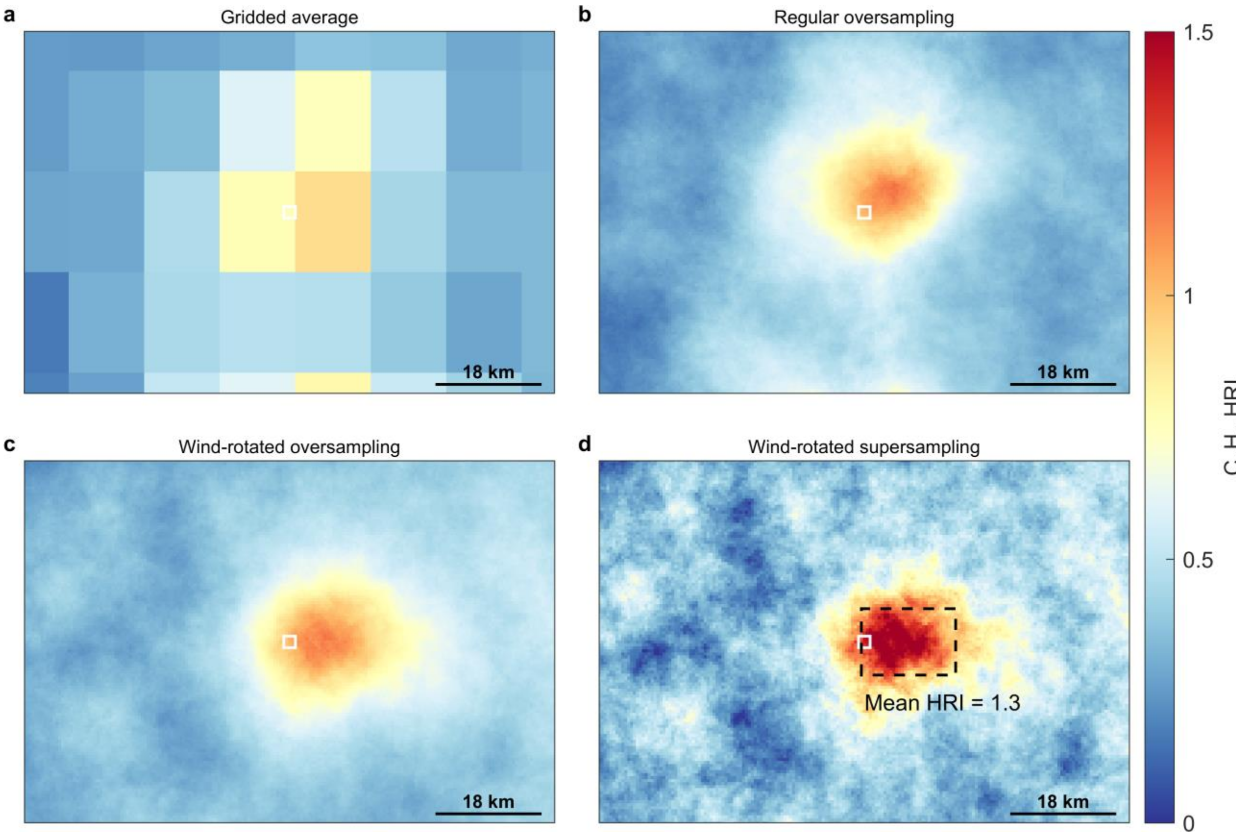

Supplementary Fig. 1 | Averaging techniques applied to the IASI C_2H_4 HRI over Mengxi Park (Inner Mongolia, China). a, Binned average on a 0.15° × 0.15° spatial resolution grid, **b,** oversampled average, **c,** wind-rotated oversampling, and **d,** wind-rotated supersampling of the IASI C_2H_4 HRI at a 0.01° × 0.01° spatial resolution, around the hotspot of Mengxi Park (Inner Mongolia, China). At the latitude of the hotspot (39.9° N), this corresponds to a grid resolution of 12.8 × 16.6 km (lon × lat) for **a**, and of 0.9 × 1.1 km for **b-d**. The coordinates of the presumed emitter, marked by a white square, is used as the wind rotation centre. In this work, the winds are realigned to the east (in the *x* direction). The area delimited by the black dashed line is used to calculate the averaged downwind HRI value. IASI, Infrared Atmospheric Sounding Interferometer; HRI, hyperspectral range index.

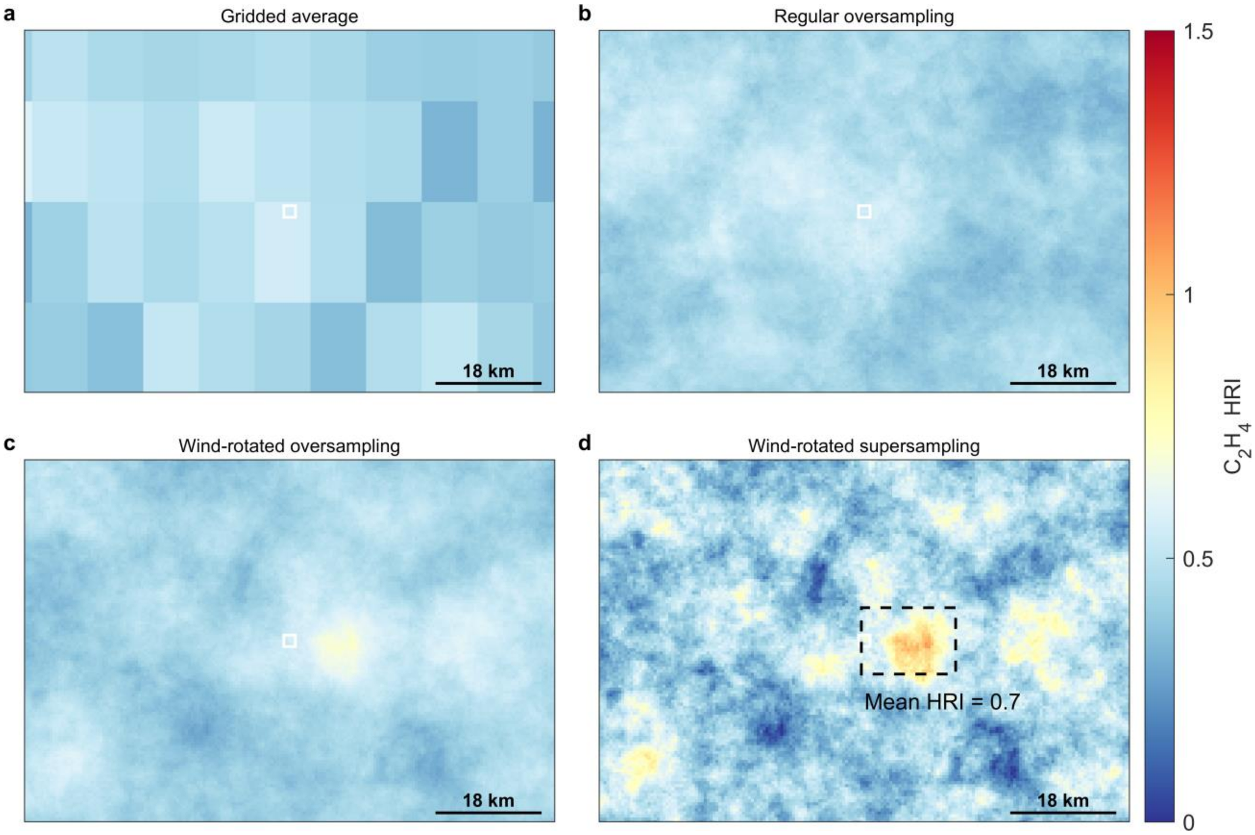

Supplementary Fig. 2 | Averaging techniques applied to the IASI C_2H_4 HRI over Novopolotsk (Belarus). **a**, Binned average on a 0.15° × 0.15° spatial resolution grid, **b,** oversampled average, **c,** wind-rotated oversampling, and **d,** wind-rotated supersampling of the IASI C_2H_4 HRI at a 0.01° × 0.01° spatial resolution, around the hotspot of Novopolotsk (Belarus). This is a typical example of a C_2H_4 hotspot that would be difficult to find without the wind-rotated supersampling. The coordinates of the presumed emitter, marked by a white square, is used as the wind rotation centre. In this work, the winds are realigned to the east (in the *x* direction). The area delimited by the black dashed line is used to calculate the averaged downwind HRI value. IASI, Infrared Atmospheric Sounding Interferometer; HRI, hyperspectral range index.

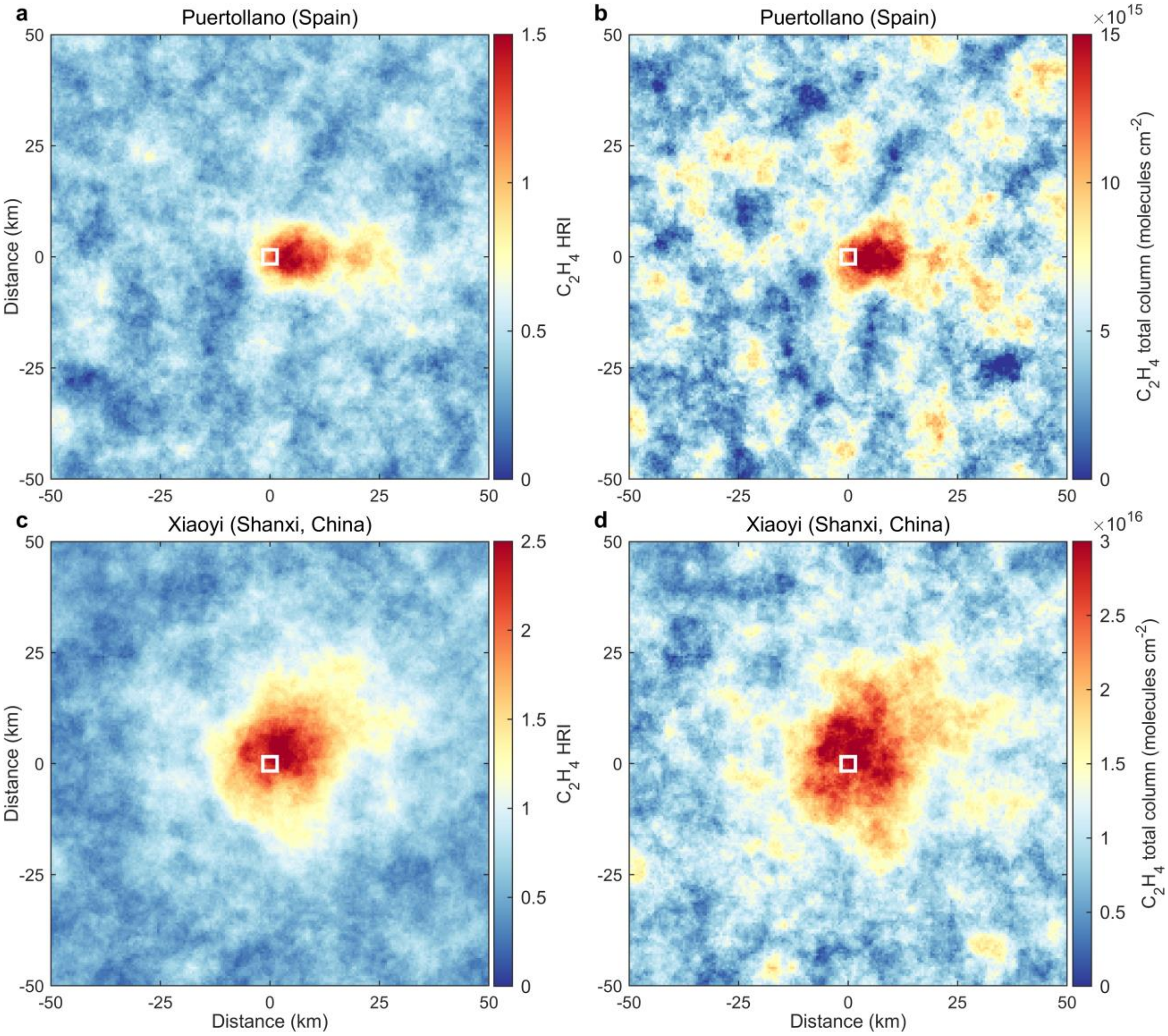

Supplementary Fig. 3 | Wind-rotated supersampling applied to C_2H_4 HRI and columns. a, c, Distribution of C_2H_4 HRI and **b, d,** columns obtained with the wind-rotated supersampling over two typical C_2H_4 hotspots. The white squares give the location of the presumed emitters used as the rotation points. In this work, the winds are realigned to the east (in the *x* direction). The column distributions exhibit a noisier background compared with the HRI. HRI, hyperspectral range index.

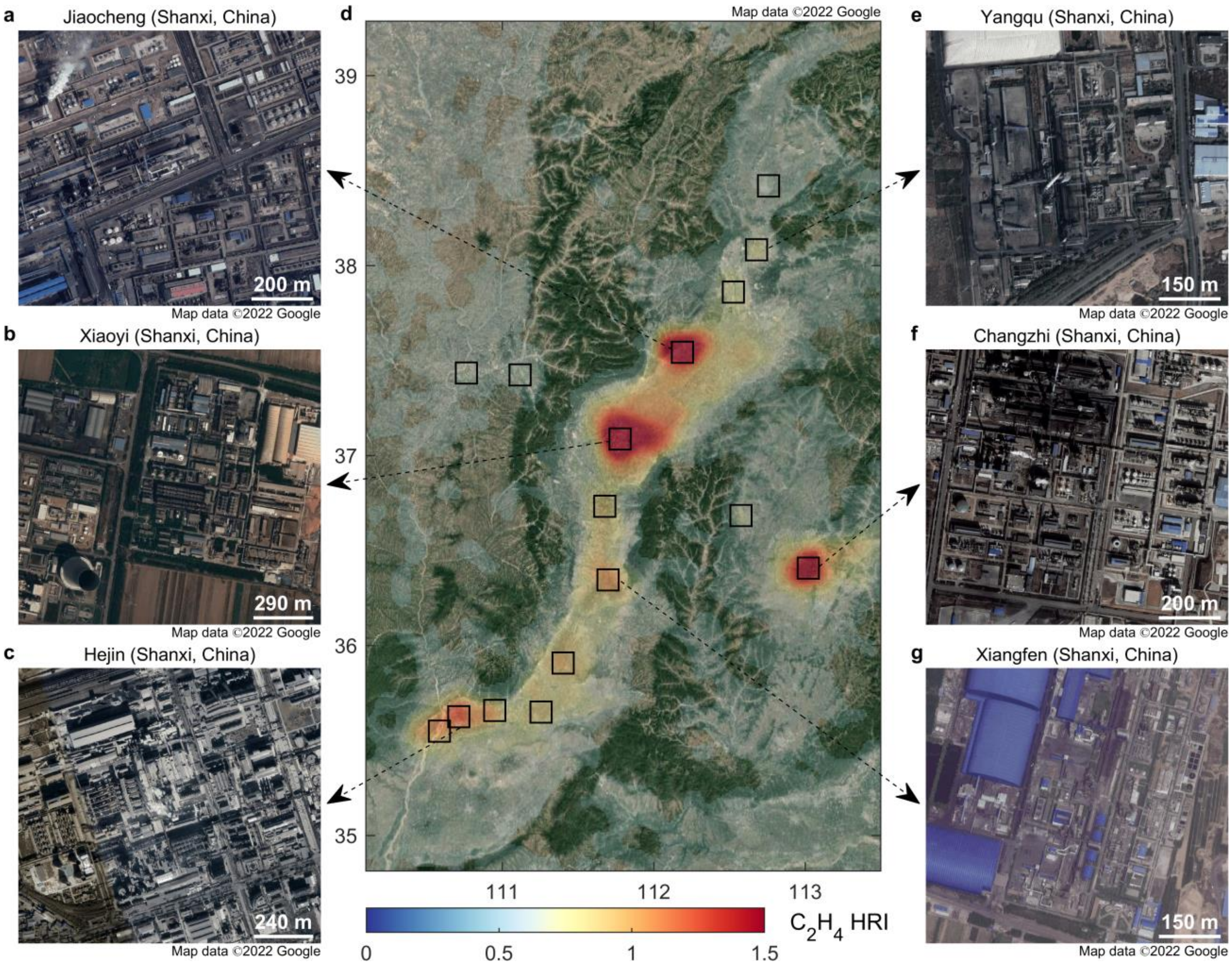

Supplementary Fig. 4 | IASI hyperfine resolution distribution, with hotspots and point-sources of ethylene. d, Zoom-in of the C_2H_4 HRI from the 13-year IASI average on satellite visible imagery, over an industrial valley of the Shanxi province, China. Hotspots of ethylene are indicated with black squares. **a-c, e-g,** Examples of close-up views on point-source emitters. IASI, Infrared Atmospheric Sounding Interferometer; HRI, hyperspectral range index. Visible imagery from Google Earth, CNES/Airbus, DigitalGlobe and Landsat/Copernicus. Map data ©2022 Google.

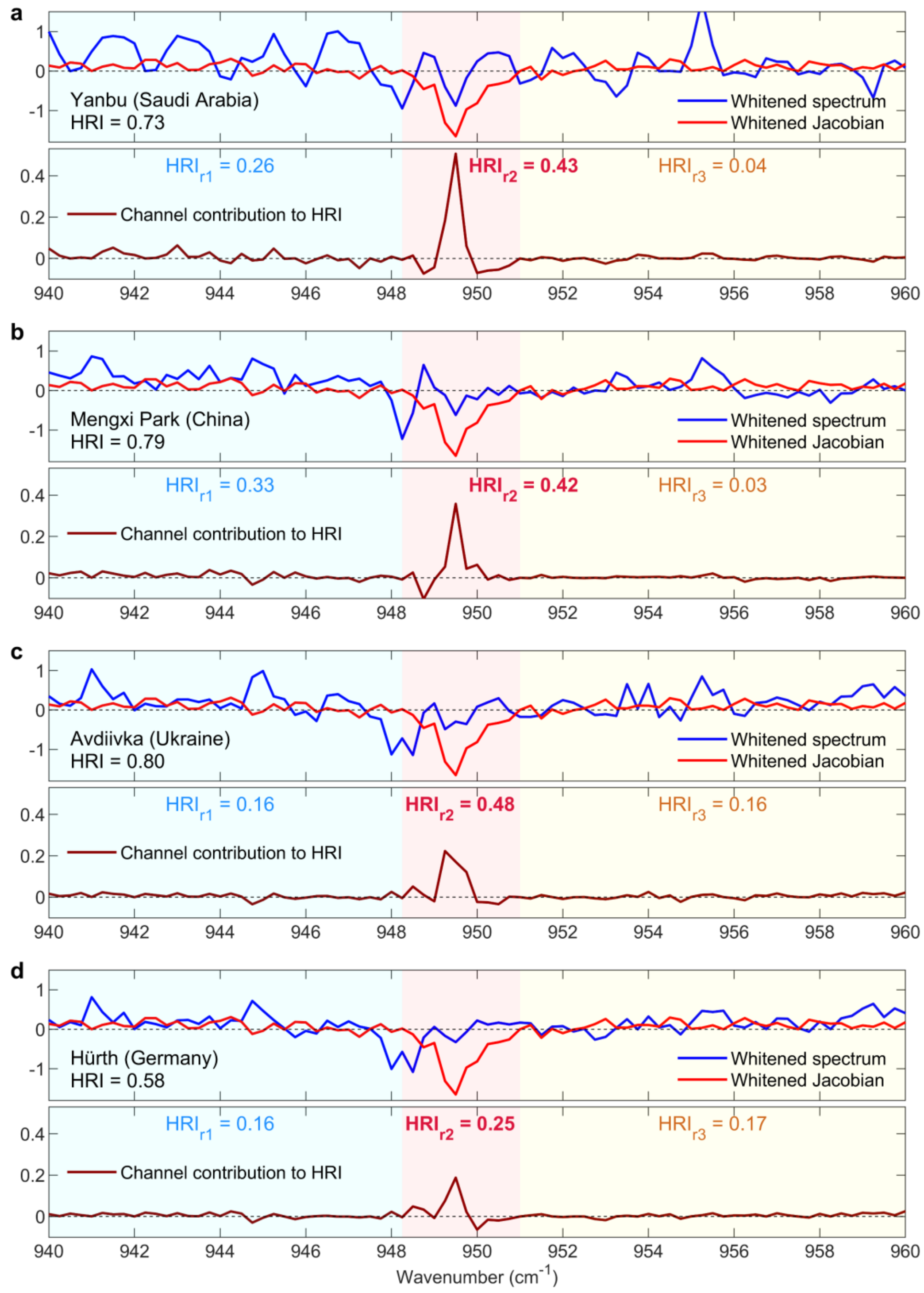

Supplementary Fig. 5 | IASI spectra analysis. a-d, Result of the whitening transformation (in blue) applied to averaged IASI spectra taken over C_2H_4 hotspots or **e-h,** regions with high HRI values, the whitened C_2H_4 Jacobian (in red), and the contribution of each channel to the HRI (in brown). The mean spectrum HRI is given in black, and the partial HRIs calculated over the spectral ranges delimited by the shaded areas ($HRI_{r1\text{-}r3}$) are in colors. The dominant partial HRI is highlighted in bold font. The location of the hotspots/regions is shown in manuscript Fig. 1. IASI, Infrared Atmospheric Sounding Interferometer; HRI, hyperspectral range index.

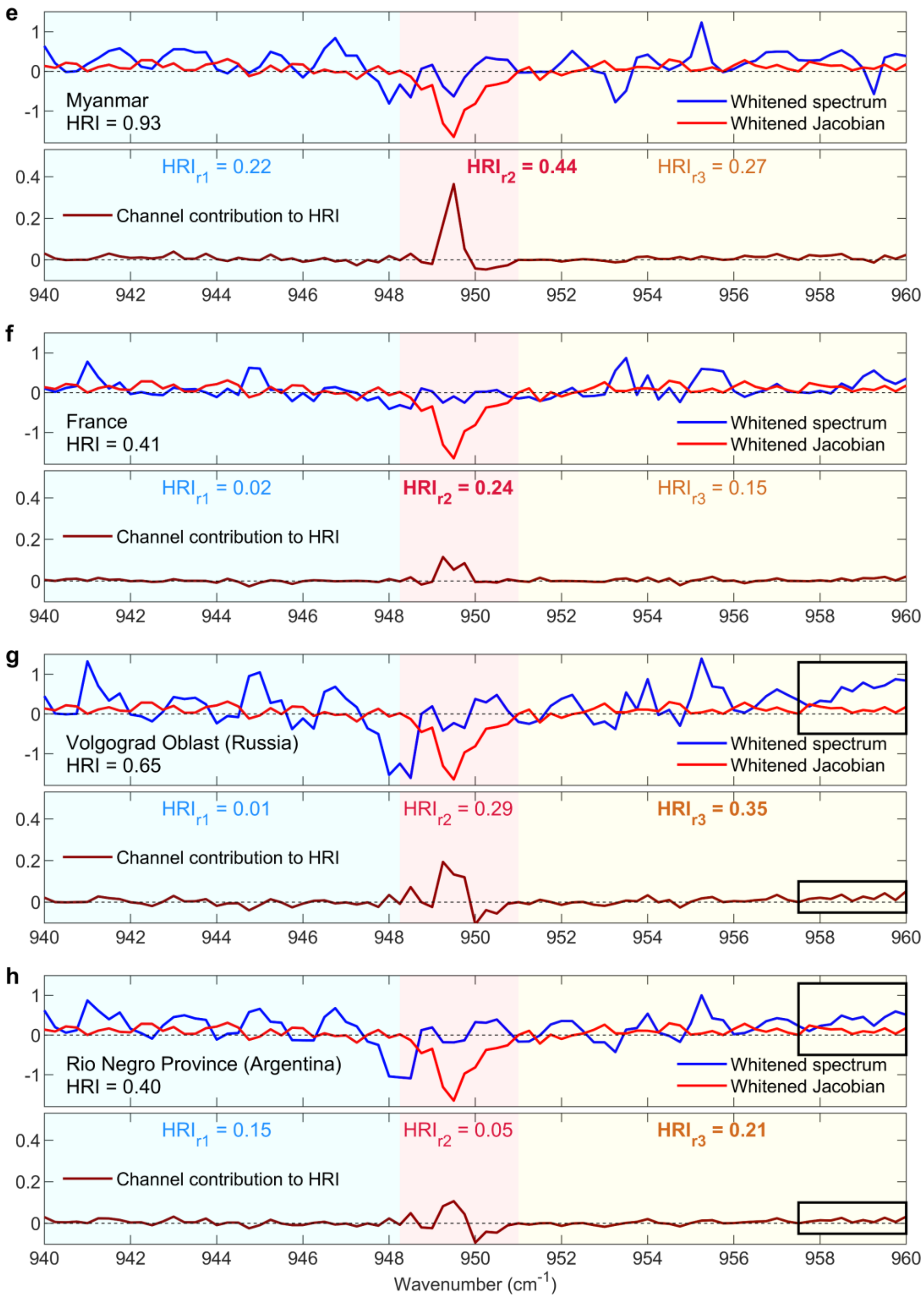

Supplementary Fig. 5 | IASI spectra analysis with whitening (continued). The black rectangle indicates wavenumbers affected by large surface emissivity features. IASI, Infrared Atmospheric Sounding Interferometer; HRI, hyperspectral range index.

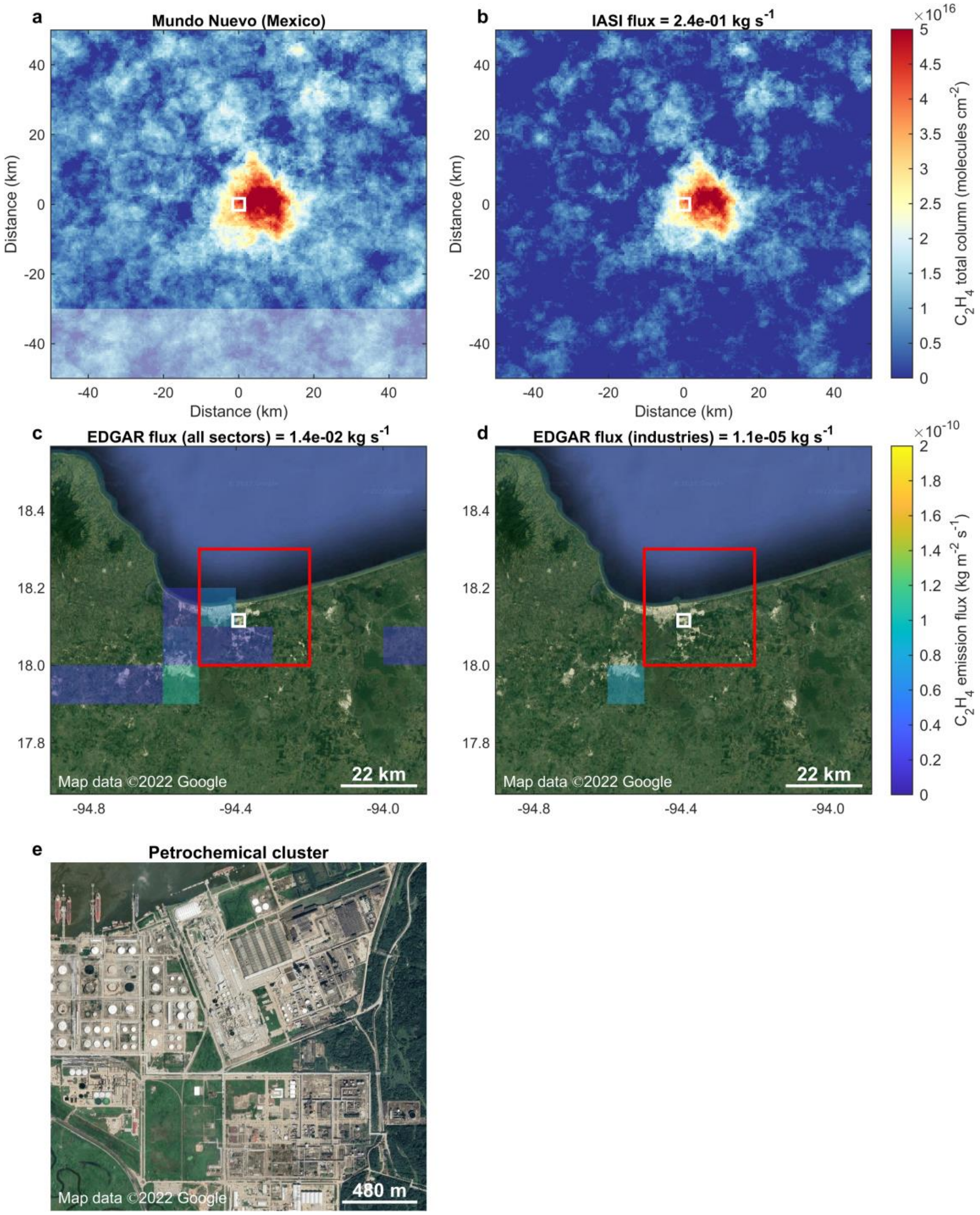

Supplementary Fig. 6 | Example of C_2H_4 flux calculation over Mundo Nuevo (Mexico). a, IASI C_2H_4 column distribution produced by the wind-rotated supersampling, with the point-source (Mundo Nuevo, Mexico) delimited in white and the area used to calculate the C_2H_4 background level shaded in semi-transparent. **b,** IASI distribution after subtracting the background column. **c,** EDGAR v4.3.2 emission fluxes of C_2H_4, over 2010-2012 and all sectors. The pixels inside the area delimited in red are used to compute the EDGAR emissions from the point-source. **d,** Same as **c**, but over the industrial sectors only. **e,** Zoom-in over the presumed emitter. IASI, Infrared Atmospheric Sounding Interferometer; EDGAR, Emission Database for Global Atmospheric Research. Visible imagery from Google Earth, CNES/Airbus, DigitalGlobe and Landsat/Copernicus. Map data ©2022 Google.

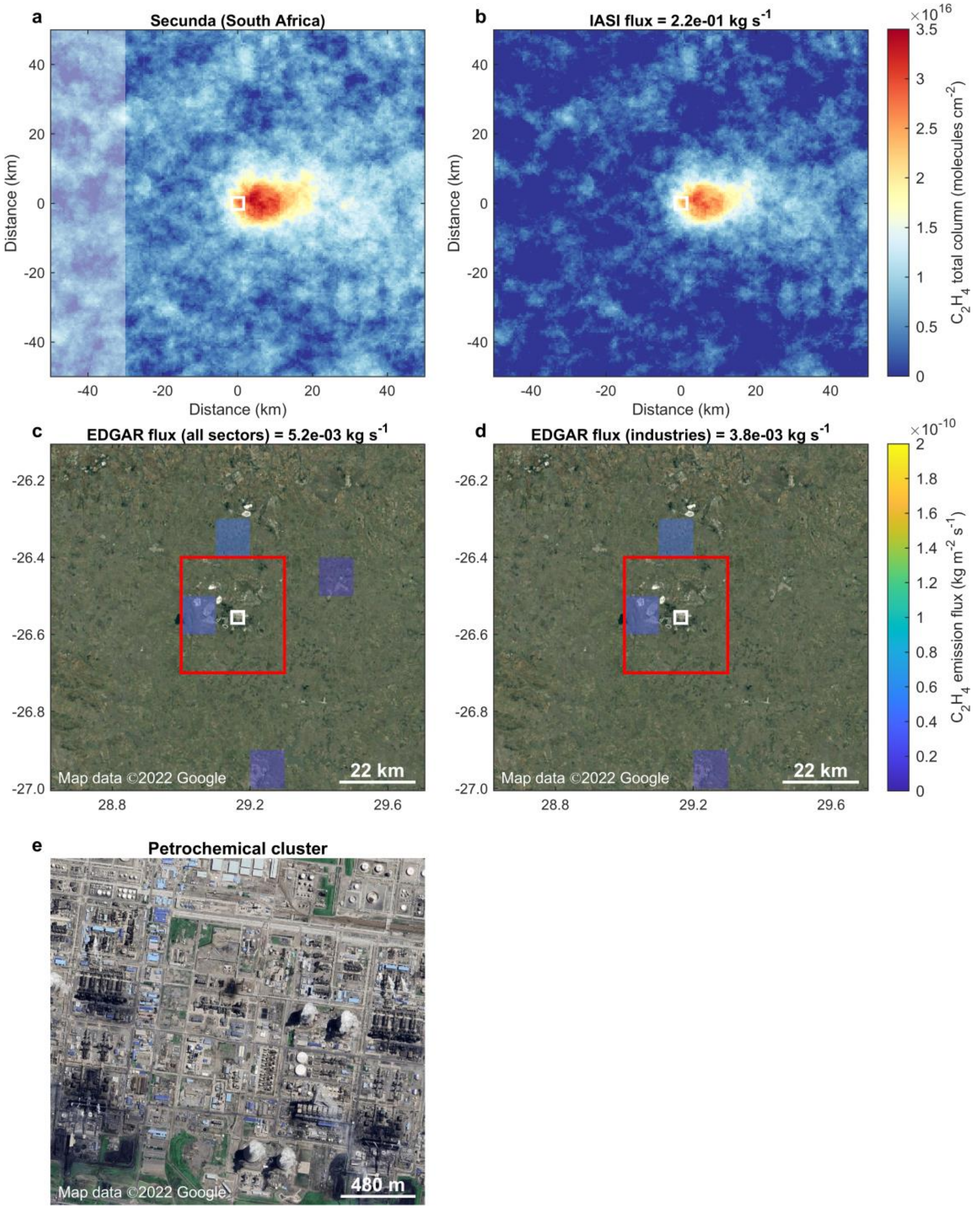

Supplementary Fig. 7 | Example of C_2H_4 flux calculation over Secunda (South Africa). a, IASI C_2H_4 column distribution produced by the wind-rotated supersampling, with the point-source (Secunda, South Africa) delimited in white and the area used to calculate the C_2H_4 background level shaded in semi-transparent. **b,** IASI distribution after subtracting the background column. **c,** EDGAR v4.3.2 emission fluxes of C_2H_4, over 2010-2012 and all sectors. The pixels inside the area delimited in red are used to compute the EDGAR emissions from the point-source. **d,** Same as **c**, but over the industrial sectors only. **e,** Zoom-in over the presumed emitter. IASI, Infrared Atmospheric Sounding Interferometer; EDGAR, Emission Database for Global Atmospheric Research. Visible imagery from Google Earth, CNES/Airbus, DigitalGlobe and Landsat/Copernicus. Map data ©2022 Google.

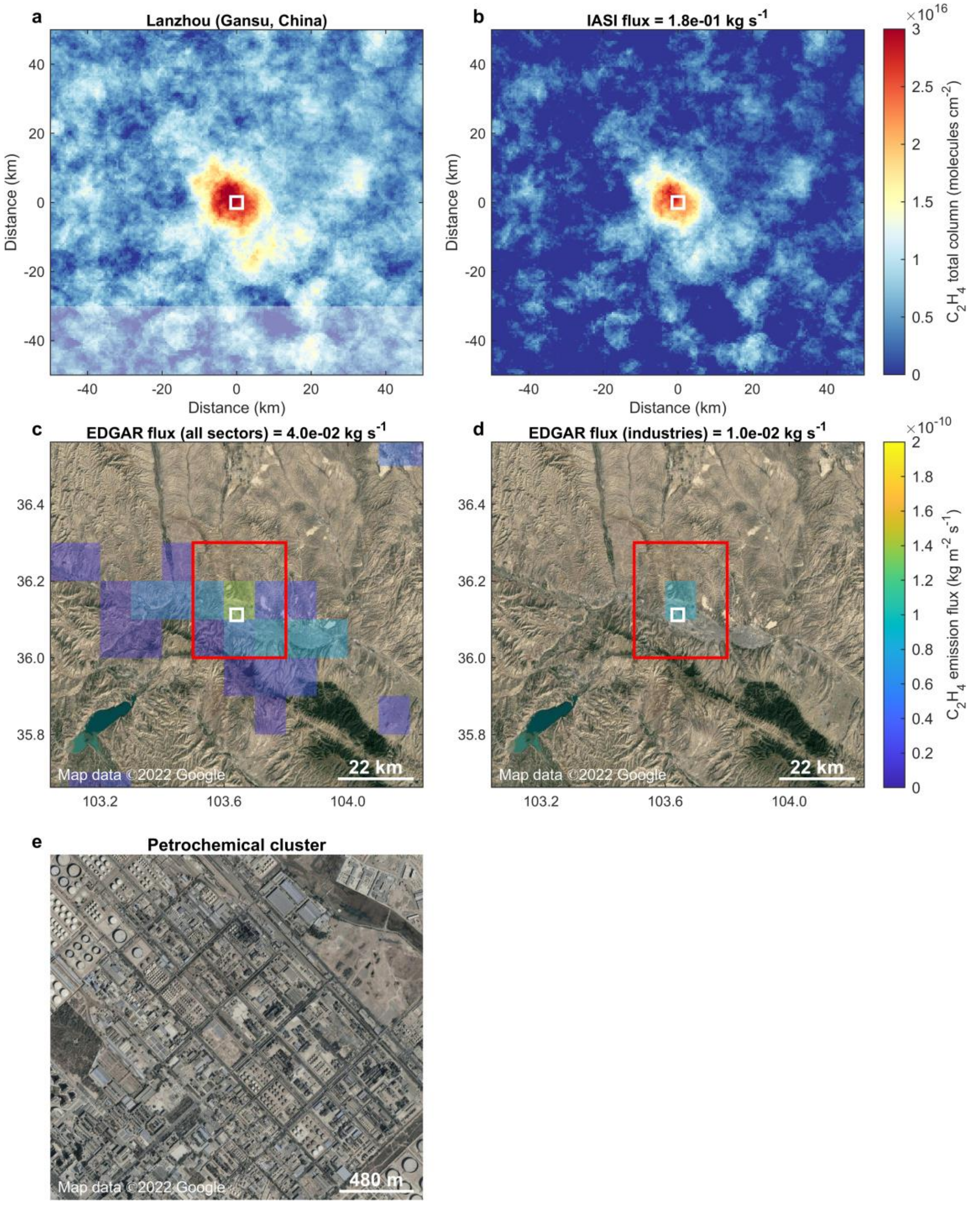

Supplementary Fig. 8 | Example of C_2H_4 flux calculation Lanzhou (Gansu, China). a, IASI C_2H_4 column distribution produced by the wind-rotated supersampling, with the point-source (Lanzhou, Gansu, China) delimited in white and the area used to calculate the C_2H_4 background level shaded in semi-transparent. **b,** IASI distribution after subtracting the background column. **c,** EDGAR v4.3.2 emission fluxes of C_2H_4, over 2010-2012 and all sectors. The pixels inside the area delimited in red are used to compute the EDGAR emissions from the point-source. **d,** Same as **c**, but over the industrial sectors only. **e,** Zoom-in over the presumed emitter. IASI, Infrared Atmospheric Sounding Interferometer; EDGAR, Emission Database for Global Atmospheric Research. Visible imagery from Google Earth, CNES/Airbus, DigitalGlobe and Landsat/Copernicus Map data ©2022 Google.

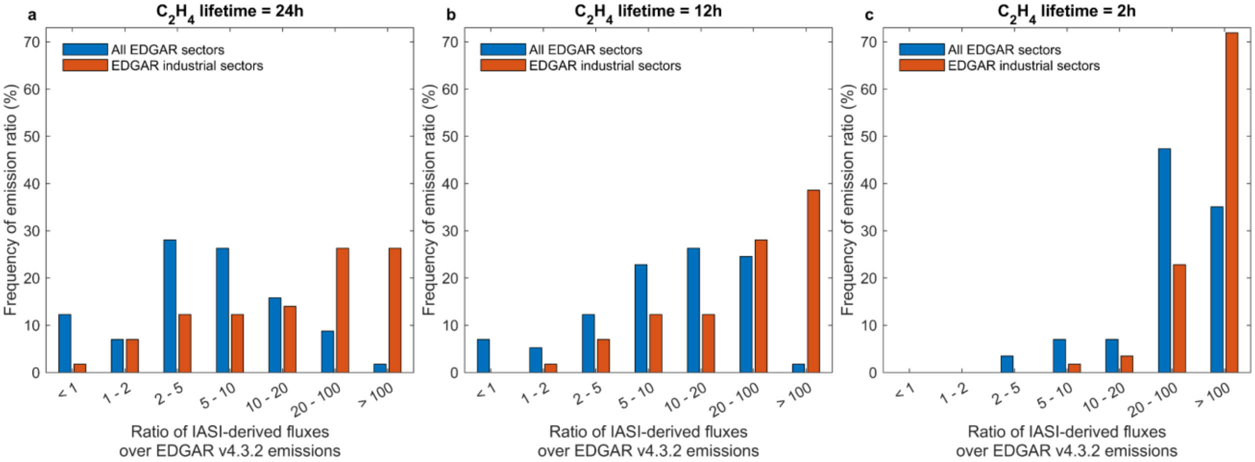

Supplementary Fig. 9 | Comparison between IASI-derived and EDGAR C_2H_4 fluxes. a, Ratio between the IASI-derived and EDGAR v4.3.2 C_2H_4 emissions, over 57 selected hotspots. EDGAR emissions were computed, respectively, for all sectors (blue) and the industrial sectors only (red). The IASI-derived fluxes were calculated assuming a C_2H_4 lifetime of 24h. **b,c,** Same as **a**, but with a C_2H_4 lifetime of 12 and 2h, respectively. IASI, Infrared Atmospheric Sounding Interferometer; EDGAR, Emission Database for Global Atmospheric Research.

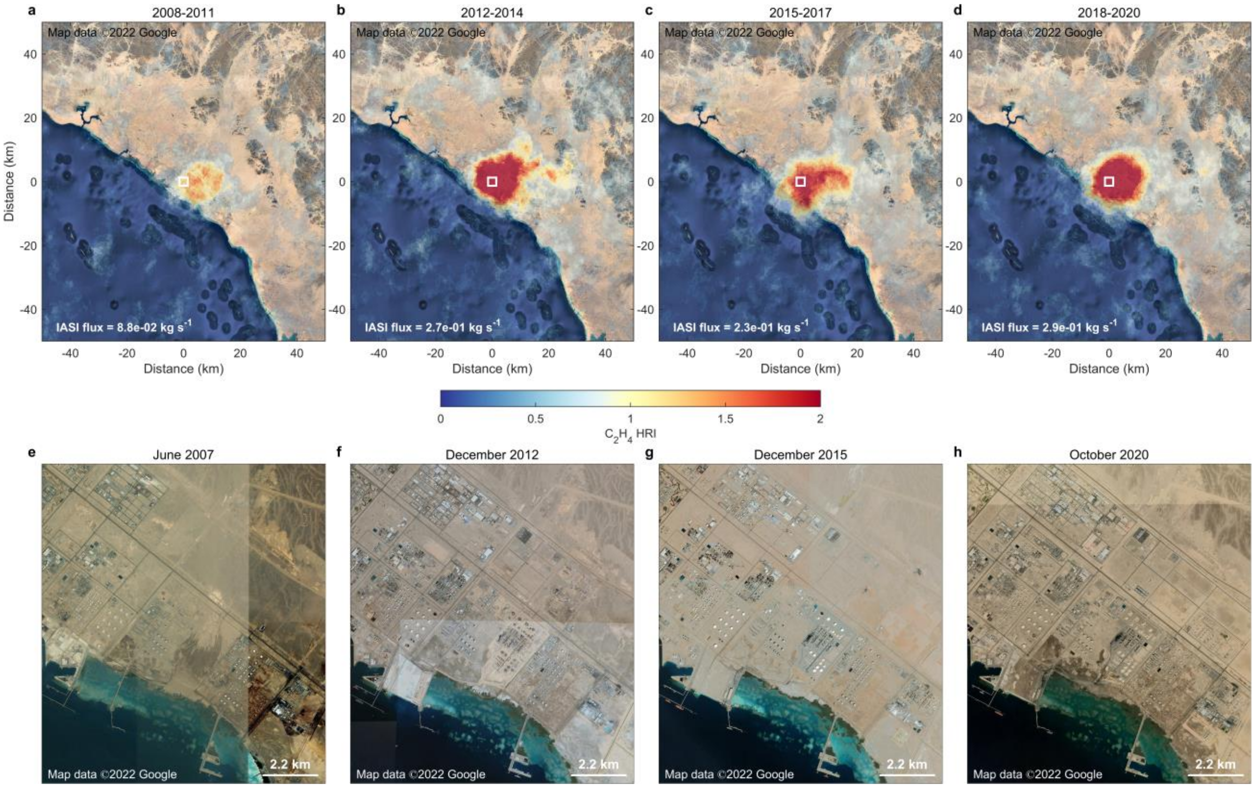

Supplementary Fig. 10 | Temporal assessment of a C_2H_4 point-source in Saudi Arabia. a-d, Wind-rotated supersampling of the IASI C_2H_4 HRI at a 0.01° × 0.01° spatial resolution, around the petrochemical hub of Yanbu (Saudi Arabia), over 2008-2011, 2012-2014, 2015-2017 and 2018-2020. The white square indicates the location of the source emitter(s). The top-down C_2H_4 emission fluxes calculated for each time period are provided in black. **e-h,** Zoom-ins on the presumed emitter(s) with satellite visible imagery taken within each time period. IASI, Infrared Atmospheric Sounding Interferometer; HRI, hyperspectral range index. Visible imagery from Google Earth, CNES/Airbus, DigitalGlobe and Landsat/Copernicus. Map data ©2022 Google.

Supplementary Table 1 | C_2H_4 point-source catalog. The categories are abbreviated as P for petrochemical and chemical industry, M for metallurgy, C for coal-related industry, U for urban, and N for non-determined. When a single C_2H_4 hotspot originates from several closely located point-sources of different types, only the two dominant categories are reported in the table (e.g., PU for both petrochemical and chemical industry, and urban categories).

Country	Name	Type	Lat	Lon
Afghanistan	Kabul	U	34.583	69.169
Algeria	El Hadjar Complex	M	36.814	7.750
Algeria	Algiers	PU	36.685	3.167
Austria	Schwechat	P	48.110	16.575
Azerbaijan	Sumgayit	P	40.519	49.580
Belarus	Novopolotsk	P	55.486	28.594
Belgium	Antwerp	P	51.277	4.200
Belgium	Geel	P	51.119	4.961
Belgium	Gent	M	51.134	3.794
Brazil	Triunfo	P	-29.877	-51.367
Brazil	São Paulo	PU	-23.624	-46.558
Brazil	Rio de Janeiro	PU	-22.851	-43.333
Brazil	Camaçari	P	-12.662	-38.329
Bulgaria	Burgas	P	42.562	27.320
Burundi	Bujumbura	U	-3.244	29.314
Canada	Vancouver	U	49.175	-123.062
Canada	Greater Sudbury	N	46.573	-81.029
Canada	Montréal	PU	45.550	-73.577
Canada	Mississauga	U	43.669	-79.664
Canada	Sarnia	P	42.911	-82.389
Chile	Santiago	U	-33.514	-70.722
Chile	La Estrella	N	-34.245	-71.605
China	Harbin	PC	45.717	126.546
China	Jilin City	PC	43.931	126.599
China	Changchun	C	43.930	125.391
China	Horqin Left Middle Banner	C	43.995	122.754
China	Benxi	M	41.259	123.626
China	Bayuquan District (Yingkou)	MC	40.285	122.185
China	Dushanzi	P	44.377	84.850
China	Fukang	C	44.091	88.355
China	Urumqi	PC	44.047	87.710
China	Hami	C	42.788	93.414
China	Yiwu	PC	43.686	94.989
China	Kuqa	C	42.113	83.090
China	Baicheng	C	41.699	81.618
China	Korla	P	41.705	86.191
China	Jiayuguan/Jiuquan	PM	39.764	98.398
China	Ulan	N	37.029	98.703
China	Baiyin	C	36.603	104.221
China	Zhongwei	C	37.568	105.153
China	Xining	M	36.626	101.797
China	Lanzhou	P	36.084	103.586
China	Maoming	P	21.566	110.947
China	Tongchuan	C	34.914	109.042
China	Baoji	N	34.359	107.186
China	Xi'an	CU	34.272	108.886
China	Pengzhou	N	30.974	103.956

Country	Name	Type	Lat	Lon
China	Chengdu	U	30.668	104.050
China	Panzhihua	M	26.522	101.705
China	Wuhan	MU	30.597	114.309
China	Ningdong Base	PC	38.267	106.562
China	Shizuishan	MC	38.922	106.425
China	Zalantun City	N	47.892	122.856
China	Hainan	MC	39.406	106.866
China	Mengxi Park	PC	39.862	106.838
China	Xiguit District	C	40.663	110.183
China	Baotou	MC	40.627	109.703
China	Tuodian Park	C	40.257	111.394
China	Jinjie Park	C	38.673	110.126
China	Sunjiacha	C	39.162	110.306
China	Lüliang	C	37.419	111.116
China	Gaohongcun	C	37.439	110.759
China	Daqing	P	46.434	125.232
China	Fushun	PC	41.839	123.902
China	Anshan	MC	41.141	122.980
China	Nanjing	PM	32.271	118.788
China	Shanghai	U	31.066	121.375
China	Shenyang	U	41.637	123.453
China	Linghai County	N	41.215	121.199
China	Tangshan	PM	39.643	118.444
China	Qian'an Steel City	M	39.907	118.531
China	Changli	M	39.694	118.868
China	Qian'an Coke Plant	C	40.091	118.799
China	Yutian	N	39.818	117.728
China	Zunhua	MC	40.196	118.026
China	Ninghe	M	39.323	117.741
China	Dagang	P	38.815	117.396
China	Xiqing District	P	39.133	117.013
China	Tianjin	U	38.997	117.332
China	Fangshan	P	39.728	115.900
China	Baoding	U	38.841	115.539
China	Dingzhou	C	38.567	114.923
China	Lingshou	N	38.284	114.449
China	Jingxing Mining District	PC	38.073	114.026
China	Shijiazhuang	PU	38.057	114.488
China	Gaocheng	C	37.965	114.939
China	Longyao	N	37.377	114.855
China	Xingtai	MC	37.174	114.527
China	Wei	N	36.926	115.296
China	Qihe	PM	36.817	116.688
China	Zibo	PC	36.807	118.151
China	Zouping	MC	36.874	117.774
China	Boxing County	P	37.050	118.328
China	Shouguang	PC	36.807	118.809
China	Jimo District	U	36.414	120.393

Country	Name	Type	Lat	Lon
China	Zhucheng	N	35.992	119.376
China	Laiwu	MC	36.333	117.574
China	Xintai	C	35.923	117.532
China	Jining	C	35.585	116.632
China	Juye County	PC	35.431	115.996
China	Tengzhou	C	34.947	117.197
China	Linyi	CU	35.041	118.300
China	Jiawang	C	34.497	117.401
China	Fengfeng Mining District	MC	36.524	114.155
China	Changzhi	MC	36.407	113.015
China	Yaoshancun	C	36.692	112.567
China	Jiaocheng	MC	37.557	112.197
China	Taiyuan	MU	37.827	112.538
China	Yangqu	C	38.081	112.683
China	Xinzhou	N	38.436	112.735
China	Xiaoyi	MC	37.093	111.793
China	Xinxiang	MC	35.289	113.961
China	Puyang	P	35.724	114.992
China	Yanzhou	C	35.554	116.854
China	Luoyang	C	34.524	112.344
China	Yanshi	C	34.731	112.762
China	Ruzhou	C	34.088	112.846
China	Shilong	C	33.867	112.921
China	Pingdingshan	N	33.774	113.451
China	Qitaihe	C	45.807	130.871
China	Houma	MC	35.656	111.264
China	Xuanhua	MC	40.593	115.035
China	Lingshi	C	36.715	111.664
China	Hongtong	PC	36.359	111.697
China	Xiangfen	MC	35.903	111.385
China	Hegang	C	47.241	130.197
China	Beiguan	C	25.322	103.840
China	Jishan	PC	35.640	110.912
China	Hejin	MC	35.636	110.715
China	Hancheng	MC	35.549	110.585
China	Renqiu	P	38.758	116.203
China	Feicheng	N	36.051	116.863
China	Liaocheng	C	36.546	115.849
China	Hefei	U	31.857	117.262
China	Jinshan	PC	30.797	121.356
Colombia	Barrancabermeja	P	7.040	-73.900
Colombia	Bogota	U	4.589	-74.132
Republic of the Congo	Pointe-Noire	P	-4.911	11.953
Czechia	Litvinov	PC	50.593	13.603
Egypt	Cairo	U	30.045	31.212
Egypt	Helwan	MU	29.826	31.313
Egypt	Suez	PM	29.954	32.402
Egypt	Alexandria	PM	31.022	29.800
Ethiopia	Addis Ababa	U	8.989	38.698
France	Le Havre	P	49.465	0.503
Germany	Hürth	PC	50.889	6.876
Germany	Duisburg	PM	51.466	6.628
Germany	Troisdorf	N	50.841	7.142
Germany	Stade	P	53.685	9.572
Germany	Ludwigshafen	P	49.546	8.365
Germany	Brake	N	53.348	8.606

Country	Name	Type	Lat	Lon
Ghana	Accra	U	5.673	-0.123
Guatemala	Obero	N	14.163	-90.922
Hungary	Tiszaújváros	P	47.863	21.036
Hungary	Dunaújváros	M	46.962	18.885
Hungary	Gyor	N	47.745	17.631
India	Koyali	P	22.358	73.111
India	Dahej	P	21.681	72.578
India	Surat	U	21.198	72.840
India	Jamnagar	P	22.294	69.886
India	Nagothana	P	18.556	73.127
India	Dolvi	M	18.753	73.044
India	Chalakudy	N	10.264	76.308
India	Salem	M	11.742	78.023
India	Hyderabad	U	17.457	78.469
India	Bhilai	M	21.174	81.410
India	Raipur	U	21.235	81.601
India	Raipur industrial area	M	21.418	81.726
India	Angul	M	20.797	85.199
India	Talcher	C	20.958	85.132
India	Kalinganagar	M	20.950	86.002
India	Raigarh	M	21.885	83.433
India	Rourkela	M	22.205	84.829
India	Jamshedpur	MC	22.790	86.153
India	Bokaro Steel City	M	23.663	86.112
India	Katras	C	23.795	86.281
India	Kolkata	U	22.635	88.196
India	Asansol	M	23.654	87.021
India	Durgapur	M	23.569	87.251
India	Visakhapatnam	MU	17.610	83.142
India	Phaphund	P	26.649	79.472
India	Panipat	P	29.469	76.901
Indonesia	Lhoknga	N	5.421	95.217
Indonesia	Medan City	U	3.591	98.695
Indonesia	Jakarta	U	-6.231	106.796
Indonesia	Bandung	U	-6.891	107.623
Indonesia	Surabaya	N	-7.460	112.665
Iran	Asalouyeh	P	27.561	52.533
Iran	Tehran	U	35.767	51.445
Iran	Isfahan	U	32.667	51.716
Iran	Zarrin Shahr	M	32.431	51.364
Iran	Bandar-e Emam Khomeyni	P	30.528	49.019
Iran	Shazand	P	34.012	49.494
Iran	Mashhad	U	36.309	59.573
Iran	Tabriz	P	38.070	46.082
Iran	Lavan	P	26.759	53.327
Iraq	Baghdad	U	33.266	44.376
Israel	Haifa	P	32.765	35.078
Italy	Bari	N	41.040	16.906
Italy	Brindisi	P	40.597	17.871
Italy	Cagliari	N	39.313	9.092
Italy	Ragusa	P	36.885	14.757
Italy	Priolo	P	37.192	15.138
Italy	Ravenna	N	44.327	12.213
Italy	Minerbio	N	44.611	11.491
Italy	Ferrara	P	44.915	11.864
Italy	Mantua	P	45.160	10.776

Country	Name	Type	Lat	Lon
Italy	Venice	N	45.584	12.335
Japan	Fukuyama	M	34.501	133.474
Japan	Kurashiki/Sakaide	PM	34.498	133.744
Japan	Okayama/Higashi Ward	N	34.612	134.041
Japan	Shunan	P	34.033	131.776
Japan	Oita	PM	33.261	131.731
Japan	Nobeoka	N	32.581	131.722
Japan	Himeji/Takasago/ Kakogawa	PM	34.729	134.760
Japan	Suma (Kobe)	N	34.668	135.135
Japan	Osaka	PM	34.782	135.519
Japan	Yokkaichi	P	34.983	136.541
Japan	Mito	U	36.328	140.451
Japan	Nagoya	PM	35.044	136.927
Japan	Tokyo Bay	PM	35.543	139.771
Japan	Kamisu/Kashima	PM	35.886	140.574
Japan	Minamisoma	C	37.701	141.004
Japan	Sendai	P	38.251	140.893
Japan	Hirosaki	U	40.606	140.448
Japan	Hakodate	U	41.790	140.716
Japan	Tomakomai	P	42.709	141.684
Japan	Sapporo	U	43.114	141.360
Kazakhstan	Tengiz Field	P	46.143	53.459
Kazakhstan	Temirtau	MC	50.016	72.984
Kuwait	Shuaiba	P	29.025	48.143
Libya	Benghazi	U	32.161	20.232
Libya	Misrata	MU	32.337	15.067
Malaysia	Kuala Lumpur	U	3.140	101.614
Mexico	Mundo Nuevo	P	18.103	-94.367
Mexico	Mexico City	U	19.433	-99.152
Mexico	Puebla	U	19.043	-98.182
Mexico	Cuautla	U	18.807	-98.913
Mexico	Guadalajara	U	20.634	-103.342
Mexico	Monterrey	MU	25.667	-100.191
Mexico	Monclova	M	26.939	-101.429
Mexico	Tijuana	U	32.505	-117.033
Morocco	Casablanca	PU	33.573	-7.505
Nigeria	Lagos	U	6.511	3.322
Nigeria	Benin City	U	6.289	5.626
North Korea	Chongjin	M	41.717	129.710
North Korea	Pyongyang	U	39.042	125.803
North Korea	Chaeryong	C	38.346	125.723
Pakistan	Karachi	PU	24.881	67.155
Pakistan	Lahore	U	31.549	74.328
Peru	Lima	U	-12.050	-76.923
Philippines	Manila	U	14.659	121.107
Poland	Ostrow Wielkopolski	N	51.631	17.752
Poland	Poznan	U	52.409	16.934
Poland	Bedzin	MC	50.367	19.109
Poland	Plock	P	52.594	19.611
Qatar	Mesaieed	P	24.933	51.534
Russia	Novokuybyshevsk	P	53.057	49.931
Russia	Budyonnovsk	P	44.830	44.109
Russia	Omsk	P	55.010	73.396
Russia	Dzerzhinsk	P	56.257	43.578
Russia	Kazan	P	55.875	49.011

Country	Name	Type	Lat	Lon
Russia	Nizhnekamsk	P	55.587	51.954
Russia	Sterlitamak	P	53.678	55.891
Russia	Salavat	P	53.415	55.865
Russia	Novotroitsk/Orsk	PM	51.220	58.405
Russia	Magnitogorsk	MC	53.409	59.025
Russia	Korsakov	P	46.614	142.891
Russia	St Petersburg	U	59.861	30.414
Russia	Moscow	PU	55.750	37.664
Russia	Perm	P	57.895	56.118
Russia	Nizhny Tagil	M	57.916	60.019
Russia	Lipetsk	M	52.649	39.455
Russia	Zima	C	54.058	102.006
Russia	Angarsk	P	52.580	103.990
Russia	Chelyabinsk	MC	55.287	61.427
Saudi Arabia	Al Jubail	PM	27.024	49.555
Saudi Arabia	Rabigh	P	22.692	39.034
Saudi Arabia	Yanbu	P	24.009	38.214
Serbia	Pancevo	P	44.853	20.671
Serbia	Novi Sad	P	45.265	19.863
Singapore	Singapore	P	1.288	103.683
Slovakia	Bratislava	P	48.080	17.149
Slovakia	Kosice	M	48.642	21.188
South Africa	Secunda	PC	-26.561	29.171
South Africa	Sasolburg	PC	-26.854	27.829
South Korea	Gwangyang/Yeosu	PM	34.810	127.691
South Korea	Ulsan	P	35.489	129.329
South Korea	Gangneung	C	37.748	128.916
South Korea	Daesan	P	36.992	126.392
South Korea	Incheon	PM	37.501	126.774
South Korea	Busan	U	35.218	129.122
Spain	Tarragona	P	41.163	1.193
Spain	Puertollano	P	38.673	-4.049
Spain	Chiva	N	39.469	-0.678
Taiwan	Kaohsiung	PU	22.718	120.312
Taiwan	Taichung	MC	24.138	120.571
Taiwan	Tainan City	U	23.009	120.245
The Netherlands	Roermond	N	51.165	5.981
The Netherlands	Terneuzen	P	51.350	3.752
The Netherlands	Geleen	P	50.987	5.827
The Netherlands	Emmen	N	52.835	6.943
The Netherlands	Amsterdam	PU	52.374	4.798
Turkey	Aliaga	P	38.748	26.958
Ukraine	Zaporizhzhia	M	47.809	35.069
Ukraine	Kryvyi Rih	MC	47.886	33.385
Ukraine	Kamianske	M	48.446	34.609
Ukraine	Alchevsk	MC	48.478	38.796
Ukraine	Yenakiieve	MC	48.220	38.227
Ukraine	Makiivka	MC	48.060	37.972
Ukraine	Mariupol	M	47.157	37.492
Ukraine	Avdiivka	C	48.171	37.699
United Kingdom	Middlesbrough	PM	54.541	-1.128
United Kingdom	Gainsborough	C	53.355	-0.795
United Kingdom	Hull	P	53.762	-0.297
United Kingdom	Ellesmere Port	P	53.332	-2.818
USA	Los Angeles	PU	34.011	-118.319
USA	San Bernardino	U	34.011	-117.628
USA	Philadelphia	PU	39.883	-75.300

Country	Name	Type	Lat	Lon
USA	Levittown	N	40.033	-74.859
USA	Newark	PU	40.646	-74.129
USA	Queens	U	40.739	-73.790
USA	Boston	U	42.383	-71.176
USA	Clinton	P	41.846	-90.260
USA	Detroit	PM	42.339	-83.245
USA	Houston	PU	29.722	-95.082
USA	Orange	P	30.066	-93.800
USA	Lake Charles	P	30.165	-93.276
USA	Beaumont	P	30.066	-94.169
USA	Baton Rouge	P	30.477	-91.173
USA	New Orleans	U	29.994	-90.022
USA	Garyville	P	30.054	-90.554
Uzbekistan	Fergana	P	40.534	71.833
Venezuela	Caracas	U	10.477	-66.811
Venezuela	Maracaibo	PU	10.667	-71.682
Vietnam	Ho Chi Minh City (Saigon)	U	10.790	106.644

Supplementary Table 2 | C_2H_4 emission fluxes. IASI-based and EDGAR (v4.3.2) emission fluxes of ethylene (in kg s^{-1}) calculated over selected hotspots. The IASI fluxes reported here were obtained assuming a C_2H_4 lifetime of 12 hours (24 hours – 2 hours). The EDGAR emissions are computed for all the sectors and for the industrial sectors only.

Hotspot	Country	IASI flux	EDGAR flux	
			All sectors	Industries
Al Jubail	Saudi Arabia	3.4 (1.7 – 20.6) × 10^{-1}	4.3 × 10^{-2}	3.2 × 10^{-2}
Aliaga	Turkey	1.1 (0.5 – 6.5) × 10^{-1}	4.3 × 10^{-3}	1.2 × 10^{-3}
Angarsk	Russia	4.4 (2.2 – 26.4) × 10^{-2}	7.1 × 10^{-3}	2.5 × 10^{-3}
Asalouyeh	Iran	1.6 (0.8 – 9.5) × 10^{-1}	3.8 × 10^{-3}	6.3 × 10^{-6}
Avdiivka	Ukraine	8.1 (4.1 – 48.5) × 10^{-2}	1.9 × 10^{-2}	1.2 × 10^{-2}
Bandar-e Emam Khomeyni	Iran	1.8 (0.9 – 10.8) × 10^{-1}	1.1 × 10^{-2}	2.1 × 10^{-6}
Baotou	China	2.0 (1.0 – 11.8) × 10^{-1}	3.5 × 10^{-2}	1.4 × 10^{-3}
Bokaro Steel City	India	1.2 (0.6 – 7.3) × 10^{-1}	3.0 × 10^{-2}	7.0 × 10^{-3}
Cairo	Egypt	1.7 (0.8 – 10.1) × 10^{-1}	8.9 × 10^{-2}	6.4 × 10^{-3}
Camaçari	Brazil	3.0 (1.5 – 18.1) × 10^{-1}	6.5 × 10^{-3}	2.1 × 10^{-3}
Changzhi	China	2.2 (1.1 – 13.0) × 10^{-1}	2.0 × 10^{-2}	8.5 × 10^{-4}
Daesan	South Korea	1.1 (0.5 – 6.5) × 10^{-1}	3.0 × 10^{-2}	3.0 × 10^{-2}
Dagang	China	1.6 (0.8 – 9.8) × 10^{-1}	2.2 × 10^{-2}	7.0 × 10^{-4}
Dahej	India	3.5 (1.7 – 20.9) × 10^{-2}	1.4 × 10^{-3}	2.7 × 10^{-4}
Dingzhou	China	5.7 (2.9 – 34.4) × 10^{-2}	2.7 × 10^{-2}	1.2 × 10^{-3}
Dolvi	India	1.4 (0.7 – 8.1) × 10^{-1}	1.1 × 10^{-2}	1.7 × 10^{-6}
Fangshan	China	9.6 (4.8 – 57.4) × 10^{-2}	1.3 × 10^{-2}	2.6 × 10^{-4}
Fengfeng Mining District	China	4.5 (2.3 – 27.3) × 10^{-1}	8.4 × 10^{-2}	7.9 × 10^{-3}
Gwangyang - Yeosu	South Korea	2.4 (1.2 – 14.3) × 10^{-1}	3.9 × 10^{-2}	3.6 × 10^{-2}
Haifa	Israel	9.0 (4.5 – 53.8) × 10^{-2}	4.5 × 10^{-3}	7.0 × 10^{-4}
Hainan	China	1.0 (0.5 – 6.1) × 10^{-1}	8.4 × 10^{-3}	3.9 × 10^{-3}
Hejin	China	3.0 (1.5 – 17.9) × 10^{-1}	1.2 × 10^{-2}	1.8 × 10^{-4}
Hongtong	China	1.7 (0.8 – 10.0) × 10^{-1}	1.0 × 10^{-2}	5.5 × 10^{-5}
Hürth	Germany	1.1 (0.6 – 6.9) × 10^{-1}	5.6 × 10^{-2}	1.4 × 10^{-2}
Jakarta	Indonesia	10.2 (5.2 – 62.4) × 10^{-1}	8.6 × 10^{-1}	1.2 × 10^{-1}
Jiaocheng	China	2.2 (1.1 – 13.5) × 10^{-1}	1.8 × 10^{-2}	8.5 × 10^{-4}
Jingxing Mining District	China	8.5 (4.2 – 50.8) × 10^{-2}	1.1 × 10^{-2}	4.8 × 10^{-4}
Kamisu - Kashima	Japan	1.5 (0.8 – 9.1) × 10^{-1}	1.4 × 10^{-2}	1.0 × 10^{-2}
Kaohsiung	Taiwan	3.1 (1.5 – 18.3) × 10^{-1}	4.9 × 10^{-2}	2.9 × 10^{-2}
Kazan	Russia	1.1 (0.6 – 6.7) × 10^{-1}	8.4 × 10^{-3}	1.2 × 10^{-3}
Koyali	India	1.3 (0.7 – 7.9) × 10^{-1}	1.2 × 10^{-1}	5.8 × 10^{-2}
Kuqa	China	5.2 (2.6 – 31.4) × 10^{-2}	6.5 × 10^{-4}	2.3 × 10^{-6}
Lanzhou	China	1.8 (0.9 – 10.5) × 10-1	4.0 × 10^{-2}	1.0 × 10^{-2}
Lavan	Iran	6.6 (3.3 – 39.7) × 10^{-2}	1.9 × 10^{-3}	1.8 × 10^{-3}
Maracaibo	Venezuela	2.0 (1.0 – 12.2) × 10^{-1}	8.7 × 10^{-2}	9.0 × 10^{-3}
Mengxi Park	China	1.5 (0.8 – 9.1) × 10^{-1}	2.6 × 10^{-3}	1.1 × 10^{-5}

Hotspot	Country	IASI flux	EDGAR flux	
			All sectors	Industries
Mexico City	Mexico	2.2 (1.1 – 13.0) × 10^{-1}	2.4 × 10^{-1}	8.1 × 10^{-3}
Mundo Nuevo	Mexico	2.4 (1.2 – 14.4) × 10^{-1}	1.4 × 10^{-2}	1.1 × 10^{-5}
Nagothana	India	1.4 (0.7 – 8.3) × 10^{-1}	8.2 × 10^{-3}	1.2 × 10^{-6}
Nanjing	China	7.5 (3.8 – 45.2) × 10^{-2}	8.6 × 10^{-2}	3.4 × 10^{-2}
Ningdong Base	China	7.0 (3.5 – 42.1) × 10^{-2}	3.2 × 10^{-3}	1.5 × 10^{-3}
Novopolotsk	Belarus	6.6 (3.3 – 39.3) × 10^{-2}	5.4 × 10^{-3}	1.5 × 10^{-3}
Puertollano	Spain	6.7 (3.3 – 40.2) × 10^{-2}	2.4 × 10^{-3}	1.6 × 10^{-3}
Rourkela	India	1.3 (0.7 – 8.0) × 10^{-1}	2.5 × 10^{-2}	3.8 × 10^{-3}
Sasolburg	South Africa	5.9 (2.9 – 35.2) × 10^{-2}	1.1 × 10^{-2}	7.8 × 10^{-3}
Secunda	South Africa	2.2 (1.1 – 13.1) × 10^{-1}	5.2 × 10^{-3}	3.8 × 10^{-3}
Sumgayit	Azerbaijan	1.7 (0.9 – 10.3) × 10^{-1}	5.1 × 10^{-3}	1.6 × 10^{-3}
Tarragona	Spain	1.7 (0.9 – 10.4) × 10^{-1}	1.0 × 10^{-2}	4.8 × 10^{-3}
Tehran	Iran	2.9 (1.5 – 17.7) × 10^{-1}	4.3 × 10^{-1}	8.0 × 10^{-2}
Tokyo Bay	Japan	7.1 (3.5 – 42.4) × 10^{-1}	1.4 × 10^{-1}	1.1 × 10^{-1}
Triunfo	Brazil	1.7 (0.9 – 10.2) × 10^{-1}	1.1 × 10^{-2}	7.3 × 10^{-3}
Urumqi	China	8.9 (4.5 – 53.6) × 10^{-2}	4.9 × 10^{-3}	7.0 × 10^{-3}
Visakhapatnam	India	6.0 (3.0 – 35.9) × 10^{-2}	9.2 × 10^{-2}	4.6 × 10^{-2}
Xiangfen	China	3.9 (1.9 – 23.3) × 10^{-1}	3.6 × 10^{-2}	2.0 × 10^{-3}
Xiaoyi	China	3.3 (1.7 – 19.9) × 10^{-1}	1.2 × 10^{-2}	9.6 × 10^{-5}
Yanbu	Saudi Arabia	2.0 (1.0 – 11.9) × 10^{-1}	3.7 × 10^{-2}	3.5 × 10^{-2}
Yiwu	China	2.5 (1.2 – 14.9) × 10^{-2}	2.0 × 10^{-5}	7.0 × 10^{-8}

Supplementary Table 3 | Comparison of IASI columns with independent measurements. Range of C_2H_4 volume mixing ratios (vmr) from surface in situ or low-altitude aircraft measurements found in the literature, their correspondence in total column, and range of total columns retrieved with IASI, for a suite of C_2H_4 hotspots. The (near-) surface vmr data were converted in total columns by assuming the same vertical distribution of C_2H_4 as the Gaussian function implemented by the ANNI v3 neural network to retrieve C_2H_4 columns from IASI (see Methods), scaled at the surface to match the vmr data. The 1976 US standard atmosphere and a planetary boundary layer height at 1 km altitude are assumed to keep this analysis straightforward and synthetic.

Hotspot	In situ vmr (ppb)	In situ columns (molecules cm^{-2})	IASI columns (molecules cm^{-2})	Reference(s)
Dagang (Tianjin, China)	2.6-33.1	$0.8\text{-}9.8 \times 10^{16}$	$1.5\text{-}2.5 \times 10^{16}$	1
Daesan (South Korea)	2.6-26.5	$0.8\text{-}7.8 \times 10^{16}$	$1.5\text{-}2.5 \times 10^{16}$	2, 3
Dushanzi (Xinjiang, China)	1.0-2.4	$0.3\text{-}0.7 \times 10^{16}$	$0.8\text{-}1.0 \times 10^{16}$	4
Fangshan (Beijing, China)	5.6	1.7×10^{16}	$2.0\text{-}3.0 \times 10^{16}$	5
Houston (Texas, USA)	1.4-10.0	$0.4\text{-}2.9 \times 10^{16}$	$1.0\text{-}2.0 \times 10^{16}$	6, 7, 8, 9
Kaohsiung (Taiwan)	6.7-19.9	$2.0\text{-}5.9 \times 10^{16}$	$2.0\text{-}3.0 \times 10^{16}$	10
Lanzhou (Gansu, China)	2.9-10.8	$0.9\text{-}3.2 \times 10^{16}$	$2.0\text{-}3.0 \times 10^{16}$	11, 12
Mexico City (Mexico)	10.0-37.0	$3.0\text{-}17.7 \times 10^{16}$	$1.0\text{-}2.0 \times 10^{16}$	13, 14
Nanjing (Jiangsu, China)	5.7	1.7×10^{16}	$2.0\text{-}3.0 \times 10^{16}$	15
Saõ Paulo (Brazil)	4.0-5.6	$1.2\text{-}1.6 \times 10^{16}$	$0.5\text{-}2.0 \times 10^{16}$	16, 17
Ulsan (South Korea)	7.8-29.1	$2.3\text{-}8.6 \times 10^{16}$	$1.5\text{-}2.5 \times 10^{16}$	18
Wuhan (Hubei, China)	7.8-15.3	$2.3\text{-}4.5 \times 10^{16}$	$1.5\text{-}3.0 \times 10^{16}$	19, 20
Yokohama (Tokyo Bay, Japan)	3.0-11.2	$0.9\text{-}3.3 \times 10^{16}$	$1.5\text{-}2.5 \times 10^{16}$	21

Supplementary References

[1] Wei, W., Wang, Y., Yang, G., Yue, L. & Cheng, S. Speciated VOCs emission estimate for a typical petrochemical manufacturing plant in China using inverse-dispersion calculation method. *Environmental Monitoring and Assessment* **190** (2018).

[2] Cho, C. *et al.* Evolution of formaldehyde (HCHO) in a plume originating from a petrochemical industry and its volatile organic compounds (VOCs) emission rate estimation. *Elementa: Science of the Anthropocene* **9** (2021).

[3] Simpson, I. J. *et al.* Characterization, sources and reactivity of volatile organic compounds (VOCs) in Seoul and surrounding regions during KORUS-AQ. *Elementa: Science of the Anthropocene* **8** (2020).

[4] Zhang, X. *et al.* Volatile Organic Compounds in a Petrochemical Region in Arid of NW China: Chemical Reactivity and Source Apportionment. *Atmosphere* **10**, 641 (2019).

[5] Wei, W. *et al.* Characterizing ozone pollution in a petrochemical industrial area in Beijing, China: a case study using a chemical reaction model. *Environmental Monitoring and Assessment* **187** (2015).

[6] de Gouw, J. A. *et al.* Airborne Measurements of Ethene from Industrial Sources Using Laser Photo-Acoustic Spectroscopy. *Environmental Science & Technology* **43**, 2437–2442 (2009).

[7] Johansson, J. K. E. *et al.* Emission measurements of alkenes, alkanes, SO2, and NO2 from stationary sources in Southeast Texas over a 5-year period using SOF and mobile DOAS. *Journal of Geophysical Research: Atmospheres* **119**, 1973–1991 (2014).

[8] Ryerson, T. B. *et al.* Effect of petrochemical industrial emissions of reactive alkenes and NOx tropospheric ozone formation in Houston, Texas. *Journal of Geophysical Research* **108** (2003).

[9] Wert, B. P. *et al.* Signatures of terminal alkene oxidation in airborne formaldehyde measurements during TexAQS 2000. *Journal of Geophysical Research: Atmospheres* **108**, 4104 (2003).

[10] Chang, C.-C., Sree, U., Lin, Y.-S. & Lo, J.-G. An examination of 7:00-9:00PM ambient air volatile organics in different seasons of Kaohsiung city, southern Taiwan. *Atmospheric Environment* **39**, 867–884 (2005).

[11] Jia, C. *et al.* Non-methane hydrocarbons (NMHCs) and their contribution to ozone formation potential in a petrochemical industrialized city, Northwest China. *Atmospheric Research* **169**, 225–236 (2016).

[12] Wu, Y. *et al.* The Characteristics of Ambient Non-Methane Hydrocarbons (NMHCs) in Lanzhou, China. *Atmosphere* **10**, 745 (2019).

[13] Altuzar, V., Tomás, S. A., Zelaya-Angel, O., Sánchez-Sinencio, F. & Arriaga, J. L. Atmospheric ethene concentrations in Mexico City: Indications of strong diurnal and seasonal dependences. *Atmospheric Environment* **39**, 5219–5225 (2005).

[14] Velasco, E. *et al.* Distribution, magnitudes, reactivities, ratios and diurnal patterns of volatile organic compounds in the Valley of Mexico during the MCMA 2002 & 2003 field campaigns. *Atmospheric Chemistry and Physics* **7**, 329–353 (2007).

[15] An, J. *et al.* Characteristics and source apportionment of VOCs measured in an industrial area of Nanjing, Yangtze River Delta, China. *Atmospheric Environment* **97**, 206–214 (2014).

[16] Alvim, D. S. *et al.* Determining VOCs Reactivity for Ozone Forming Potential in the Megacity of São Paulo. *Aerosol and Air Quality Research* **18**, 2460–2474 (2018).

[17] Dominutti, P. A., Nogueira, T., Borbon, A., de Fatima Andrade, M. & Fornaro, A. One-year of NMHCs hourly observations in São Paulo megacity: meteorological and traffic emissions effects in a large ethanol burning context. *Atmospheric Environment* **142**, 371–382 (2016).

[18] Na, K., Kim, Y. P., Moon, K.-C., Moon, I. & Fung, K. Concentrations of volatile organic compounds in an industrial area of Korea. *Atmospheric Environment* **35**, 2747–2756 (2001).

[19] Shen, L. *et al.* Sources Profiles of Volatile Organic Compounds (VOCs) Measured in a Typical Industrial Process in Wuhan, Central China. *Atmosphere* **9**, 297 (2018).

[20] Zheng, H. *et al.* Compositions, sources and health risks of ambient volatile organic compounds (VOCs) at a petrochemical industrial park along the Yangtze River. *Science of The Total Environment* **703**, 135505 (2020).

[21] Tiwari, V., Hanai, Y. & Masunaga, S. Ambient levels of volatile organic compounds in the vicinity of petrochemical industrial area of Yokohama, Japan. *Air Quality, Atmosphere & Health* **3**, 65–75 (2009).
